# Growth Factors Serum Levels in Coronary Artery Disease Patients Scheduled for Bypass Surgery: Perioperative Dynamics and Comparisons with Healthy Volunteers

**DOI:** 10.1155/2013/985404

**Published:** 2013-07-29

**Authors:** Inga Karu, Joel Starkopf, Kersti Zilmer, Mihkel Zilmer

**Affiliations:** ^1^North Estonia Medical Centre, Clinic of Anaesthesiology, Sütiste 19, 13419 Tallinn, Estonia; ^2^Department of Anaesthesiology and Intensive Care, University of Tartu, Puusepa 1a, 50406 Tartu, Estonia; ^3^Institute of Biochemistry, Centre of Excellence for Translational Medicine, University of Tartu, Ravila 19, 50411 Tartu, Estonia

## Abstract

*Background*. Vascular endothelial growth factors are important mediators for neovascularization of chronically ischemic adult heart, but their elevated values have also been connected with acute ischemia. Coronary artery bypass grafting (CABG) is associated with activation of inflammatory processes. We aimed to clarify whether the latter is also accompanied with acute changes in concentrations of vascular growth factors. *Methods*. Concentrations of growth factors VEGF and EGF, monocyte chemoattractant protein-1 (MCP-1), and a set of cytokines of 39 patients with stable coronary artery disease (CAD) were evaluated before and after CABG. Preoperative values were compared with data of healthy volunteers. *Results*. In comparison with CAD patients, healthy controls had significantly higher values of VEGF (15.5 (10.05–35.3) and 119.4 (55.7–136.9) pg/mL, resp.), EGF (1.70 (1.14–3.18) and 37.3 (27.1–51.9) pg/mL, resp.), and MCP-1 (111.6 (81.75–171.9) and 156.9 (134.7–241.3) pg/mL, resp.). MCP-1, but not others, demonstrated a significant rise throughout the postoperative period. Proinflammatory interleukin-6 was significantly higher and anti-inflammatory IL-4 and IL-10 lower in patients with CAD. *Conclusions*. Patients with stable CAD have lower serum levels of growth factors than healthy volunteers. MCP-1, but not VEGF and EGF, becomes elevated immediately after CABG. Inflammatory status of CAD patients was drifted towards proinflammatory state.

## 1. Introduction

Vascular endothelial growth factors are major angiogenic molecules controlling vascular growth and function, vascular homeostasis, permeability, and vasodilatation. They have been shown to be important for neovascularization of the chronically ischemic adult heart and therefore have been regarded as potential new treatment agents for ischemic heart and peripheral vascular disease [1]. Acute ischemia during coronary artery bypass grafting (CABG) surgery has been connected with a rise of the circulating level of vascular endothelial growth factor (VEGF), which might be beneficial due to stimulation of DNA synthesis both in human coronary arteries and aortic endothelial cells [2]. Epidermal growth factor (EGF) being another cytoprotective and proangiogenic growth factor has been shown to afford myocardial protection from acute stress caused by low-flow ischemia in mice [3]. Serum levels of VGEF are tightly regulated by monocyte chemoattractant protein (MCP)-1, a member of the C-C chemokine family. MCP-1 is produced by monocytes/macrophages, smooth muscle cells, and endothelial cells within atherosclerotic plaques [4]. Plasma levels of MCP-1 are positively correlated with most cardiovascular risk factors, with measures of coronary atherosclerosis burden, and with the incident coronary and peripheral artery disease [5–7].

It is well demonstrated that CABG is associated with significant activation of the inflammatory processes of the whole body. It is not ultimately clear whether this is accompanied by acute changes in concentrations of vascular growth factors. The present study was undertaken to give our contribution to this topic via using special high-sensitive chips technology measuring VEGF, EGF, and MCP-1 as well as a spectrum of serum cytokines and chemokines-interleukins-1*α* and 1*β* (IL-1*α*, *β*), interleukin-2 (IL-2), interleukin-4 (IL-4), interleukin-6 (IL-6), interleukin-8 (IL-8), interleukin-10 (IL-10), tumor necrosis factor *α* (TNF-*α*), and interferon *γ* (IFN-*γ*) in patients with stable coronary artery disease preoperatively and for two days after CABG surgery. Additionally, we compared preoperative concentrations of these markers in patients with coronary artery disease (CAD) to the data of age and sex matched healthy volunteers, to further assess changes caused by CAD.

## 2. Materials and Methods

The investigation conforms to the principles outlined in the Declaration of Helsinki. The study design was approved by the Ethics Review Committee on Human Research of the University of Tartu and the informed consent was signed the day before surgery. Patients with CAD were age and sex matched with data of healthy volunteers from the endemic reference database (courtesy of Institute of Biochemistry, University of Tartu). 

### 2.1. Coronary Artery Bypass Grafting

Standardized intravenous anesthesia (midazolam, fentanyl, propofol, pipecuronium) was used in all cases. Cardiopulmonary bypass was performed with a roller pump (Maquet Critical Care AB, Solna, Sweden) and a membrane oxygenator (Maquet Quatrox-I, Hirrlingen, Germany) under mild hypothermia (nasopharyngeal temperature 35–36°C). Warm blood cardioplegia was given antegradely into the aortic root. 

### 2.2. Biochemical Markers

Blood was sampled before induction of anesthesia (data for comparisons with healthy volunteers and perioperative dynamics baseline) and 1 h, 6 h, 18 h (1st postoperative morning) and 40 h (2nd postoperative morning), after declamping the aorta. Blood was centrifuged immediately after sampling and serum stored at −80°C until analyses.

The cytokines and growth factors were measured in sera with the Evidence Investigator Cytokine & Growth factors high-sensitivity array (CTK HS Cat. No. EV 3623; RANDOX Laboratories Ltd. Crumlin, UK) according to the manufacturer's protocol. Assay sensitivity varied from 0.12 pg/L to 2.12 pg/L depending on specific marker analyte. The reproducibility of the assay for individual cytokine was determined using the quality controls provided with the kit.

### 2.3. Statistical Analysis

Patient data were compared with Fisher's exact test. To evaluate differences between groups for serum concentrations of biochemical markers, Mann-Whitney *U *test was applied and results are referred to as median (interquartile range). Postoperative values were compared against preoperative baseline with nonparametric repeated measures (Friedman) ANOVA. In case of significant differences, Wilcoxon Matched Pairs test was applied. A value of *P* < 0.05 was assumed to be statistically significant.

## 3. Results

### 3.1. Patients

Data of 39 patients with triple-vessel CAD and 39 healthy controls were analyzed. Patient characteristics are given in Table 1. Most of CAD patients had some kind of medications or their combinations while in healthy volunteers group anybody used neither cardiac nor other medications. All of them also had normal values of routine biochemical cardiovascular risk markers (cholesterol, triglyceride, etc).

### 3.2. Vascular Growth Factors and MCP-1

**Figure 1:**
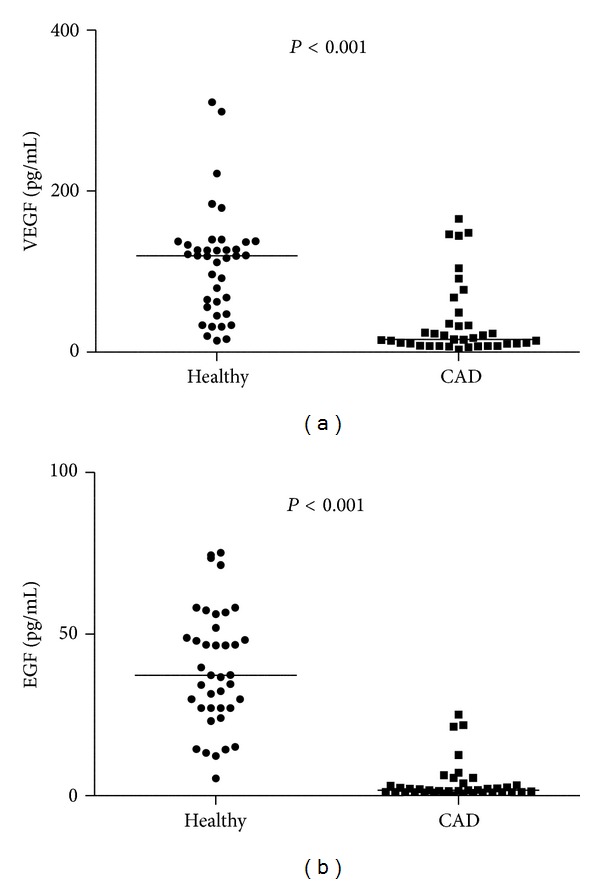
Values of VEGF (a) and EGF (b) of healthy volunteers and coronary artery disease (CAD) patients represented as single patient values with a sample median.

VEGF was significantly higher in healthy controls (119.4 (55.7–136.9) pg/mL) than in CAD patients (15.5 (10.1–35.3) pg/mL, *P* < 0.001). EGF showed a similar profile (37.3 (27.1–51.9) and 1.70 (1.14–3.18) pg/mL, resp., *P* < 0.001). Both markers showed also greater variability in healthy population (Figure 1). MCP-1 was lower in patients with CAD (111.6 (81.8–171.9) and 156.9 (134.7–241.3) pg/mL, resp., *P* < 0.001) (Figure 3). In subgroup analysis, no sex-dependent differences were found. 

**Figure 2:**
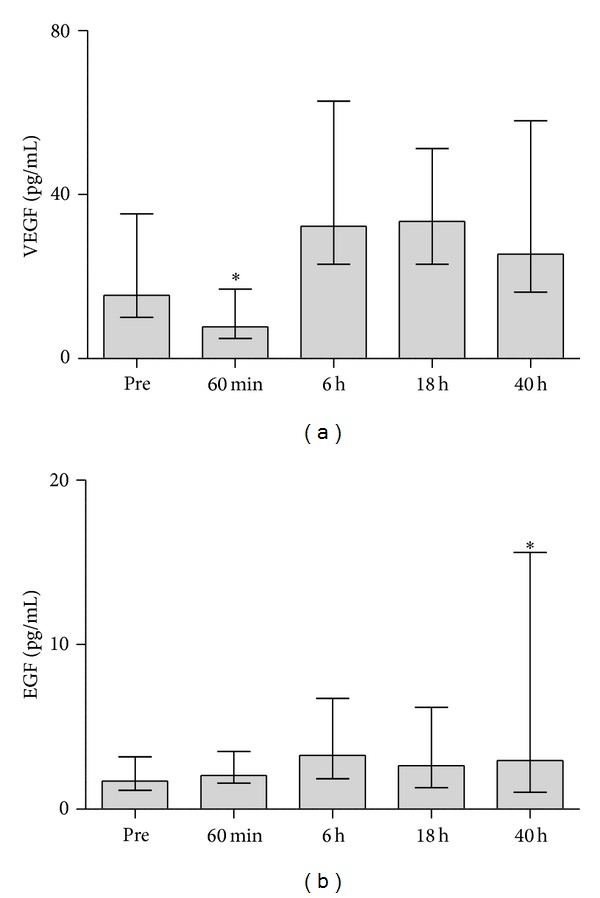
Concentrations of VEGF (a) and EGF (b) preoperatively (pre) and at time-points after declamping the aorta during CABG. Significant differences are shown in comparison with preoperative baseline. *P < 0.05. Values are presented as median with interquartile range.

**Figure 3:**
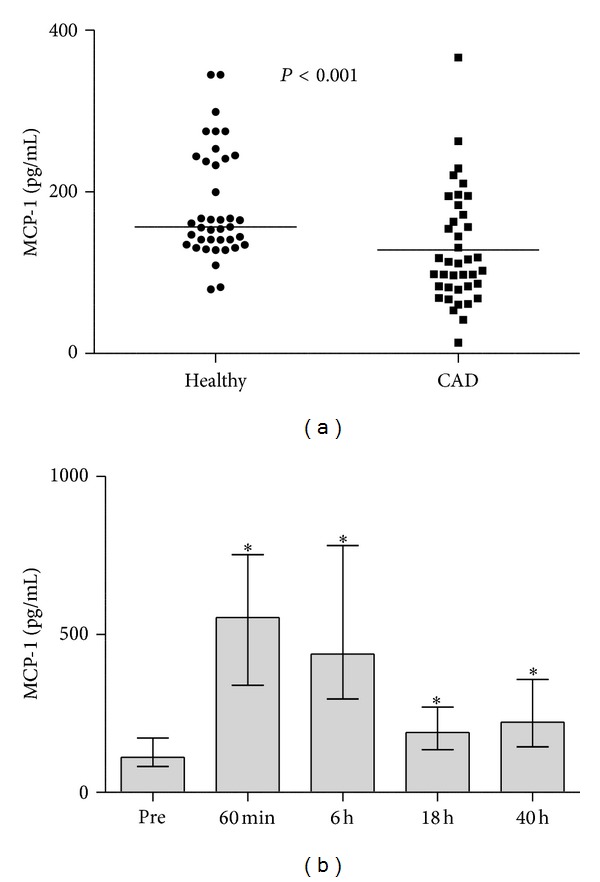
Values of MCP-1 in healthy volunteers and coronary artery disease (CAD) patients. (a) Values are represented as single patient values with a sample median. (b) Preoperative values (pre) and concentrations at time-points after declamping the aorta during CABG. Significant differences are shown in comparison with preoperative baseline. *P < 0.05. Values are presented as median with interquartile range.

Postoperative dynamics of these parameters is shown on Figures 2 and 3. 

### 3.3. Cytokines

Baseline concentrations of *proinflammatory cytokine* IL-6 was significantly higher in patients with coronary artery disease. Contrary, *antiinflammatory* IL-4 and IL-10 were lower in this group. Concentrations of other cytokines did not differ between CAD-patients and healthy controls (Table 2). 

## 4. Discussion

By using Cytokine & Growth factors high-sensitivity array technology our study demonstrates that patients with stable coronary artery disease have significantly lower serum levels of vascular growth factors VGEF and EGF as well as of MCP-1 than healthy volunteers. At the same time, they have higher level of pro-inflammatory IL-6 and lower levels of antiinflammatory IL-4 and IL-10. 

It remains difficult to determine whether observed changes in growth factors are detrimental or beneficial for the patient. On the one hand, observation of levels lower than in healthy controls would suggest that higher levels of growth factors could be desired to promote reparative processes of diseased vessels. On the other hand, associations of growth factors with inflammatory processes would allow us to speculate that observed changes, perhaps as a result of antiatherosclerotic effect of applied medications, are beneficial in long term.

VEGF has potent angiogenic, mitogenic, and vascular permeability-enhancing activities specific for endothelial cells. *In vivo*, VEGF acts directly on the endothelium and can induce angiogenesis as well as increase microvascular permeability. Based on its *in vitro* and *in vivo* properties, VEGF is expected to play important roles in inflammation. However, VEGFs also take part in pathological states by inducing microvessel growth, for example, in tumors and atherosclerotic lesions [1]. Thus, extremely high levels of VGEF would suggest excessive inflammation, while too low levels could be a sign of insufficient level of vascular repair. 

Similar to VEGF, also EGF induces development of epithelium and promotes angiogenesis. Sites of action of EGF are vascular smooth muscle and endothelial cells, and its receptors have been identified on intimal smooth muscle cells within human atherosclerotic plaque [8]. VEGF and EGF also attract monocytes and are involved in progression of atherosclerosis.

MCP-1 is another major chemoattractant, activator for monocytes and macrophages, which plays a crucial role both in the initiation and progression of atherosclerosis. Migration of blood monocytes into the arterial subendothelium is one of the important early steps in atherogenesis [9]. MCP-1 seems to be a reliable indicator of atherosclerotic plaque burden [10]. The observation that CAD patients had lower MCP-1 levels than healthy matched controls is therefore surprising. 

And there the question remains—are the levels of not only MCP-1 but also VEGF and EGF lower in CAD patients because of their concomitant medications? Statins and angiotensin converting enzyme (ACE) inhibitors have been shown to reduce these levels, and have been preoperatively taken by more than 70% of our patients. Statins have also been shown to exert antiinflammatory action [11] and patients treated with statins have shown lower postoperative VEGF levels previously [12]. Angiotensin II, as a pro-inflammatory mediator, can elicit VCAM-1 and MCP-1 expression by endothelial cells and IL-6 production by smooth muscle cells [13]. ACE-inhibitors may block angiotensin II induced MCP-1 expression by endothelial cells and IL-6 production by smooth muscle cells. Regarding the MCP-1 values in healthy volunteers, it should be emphasized that MCP-1 has not only cardiovascular but also evidently much broader background. Recent and ongoing research refers to the role of MCP-1 in various allergic conditions, immunodeficiency diseases, bone remodelling, permeability of blood-brain barrier, atherosclerosis, nephropathies, and so forth [14].

Available few reports have shown that cardiopulmonary bypass is associated with postoperative rise of growth factors [15, 16]. Myocardial ischemia secondary to cardiopulmonary bypass has been shown to be a potent stimulator of VEGF production [2] increasing the levels for up to six days after CABG [17]. We did not find a postoperative rise of VEGF or EGF in this study, but MCP-1 was raised significantly through the postoperative period. As pro-inflammatory nuclear factor kappa B transcription factor is a key mediator of MCP-1 stimulation [18], high MCP-1 levels are the expected finding after a potent stress of ischemia and reperfusion.

As expected, concentration of a major proinflammatory cytokine IL-6 was significantly higher, and at the same time antiinflammatory IL-4 and IL-10 were much lower in CAD patients than in healthy volunteers. IL-6 is a multifunctional cytokine regulating humoural and cellular responses and playing a central role in inflammation and tissue injury. Large-scale human genetic and biomarker data are consistent with a causal association between IL-6 receptor-related pathways and coronary heart disease [19]. A number of reports have described high IL-6 levels in acute coronary syndromes indicating evolving role of inflammatory markers in myocardial ischemiareperfusion. IL-6 induces the expression of VEGF, but six weeks of treatment with statins has shown to result in a significant decrease of VEGF and decrease in stimulatory effect of IL-6 [20]. In our study healthy controls had significantly higher values and more variability in concentrations of VEGF and EGF than CAD patients, but values of both groups still fell into the reference interval established from the healthy population of the STANISLAS cohort [21]. 

## 5. Conclusion

Patients with stable coronary artery disease have inflammatory status drifted towards proinflammatory state. They also have significantly lower serum levels of growth factors VGEF and EGF and of MCP-1 than healthy volunteers, but whether these changes are induced by atherosclerosis, coronary artery disease and following coronary surgery remain difficult to clarify in a clinical setting.

## Figures and Tables

**Table 1:** Characteristics of study groups. Values are provided as incidence (%) or mean (SD).

Variable	Healthy (*n* = 39)	CAD patients (*n* = 39)	*P* value
Age, years (SD)	62 (4)	64 (8)	ns
Gender (male/female)	27/12	27/12	ns
Medications*			
Ca-channel blockers, *n* (%)	—	8 (21)	
Nitrates, *n* (%)	—	23 (60)	
*β*-Blockers, *n* (%)	—	37 (94)	
ACE-inhibitors, *n* (%)	—	28 (72)	
Statins, *n* (%)	—	31 (79)	

CAD: coronary artery disease, ACE: angiotensin converting enzyme.

*All medications except salicylates were allowed up to the morning of surgery.

**Table 2:** Concentrations of cytokines in patients with 3-vessel coronary artery disease (CAD) and healthy volunteers. Values are presented as median (interquartile range).

	CAD patients (*n* = 39)	Healthy volunteers (*n* = 39)	*P* value
IL-1*α* (pg/mL)	0.12 (0.07–0.21)	0.10 (0.07–0.21)	ns
IL-1*β* (pg/mL)	0.66 (0.48–0.97)	0.66 (0.47–0.93)	ns
IL-2 (pg/mL)	1.67 (1.29–2.38)	1.67 (1.10–2.36)	ns
IL-4 (pg/mL)	1.20 (1.00–1.42)	1.38 (1.09–1.78)	0.03
IL-6 (pg/mL)	1.98 (1.02–3.39)	0.73 (0.57–1.00)	<0.001
IL-8 (pg/mL)	6.03 (4.01–7.53)	6.63 (5.13–8.04)	ns
IL-10 (pg/mL)	0.49 (0.42–0.68)	0.64 (0.51–0.80)	<0.01
TNF*α* (pg/mL)	2.66 (2.07–3.84)	2.89 (2.49–3.86)	ns
IFN*γ* (pg/mL)	1.36 (0.72–2.91)	1.37 (1.24–1.74)	ns
